# Cocaine Hydrochlorate Anæsthesia

**Published:** 1885-01

**Authors:** A. A. Hubbell

**Affiliations:** Professor of Diseases of the Eye, Ear and Throat, in the Medical Department of Niagara University


BUFFALO

### Medical and Surgical Journal.

Vol. XXIV. JANUARY, 1885. No. 6.

### Original Communications.

### Cocaine Hydrochlorate Anæsthesia.

By A. A. Hubbell, M. D.,

Professor of Diseases of the Eye, Ear and Throat, in the Medical Department of Niagara University.

The discovery that a solution of cocaine hydrochlorate applied to certain parts of the body will render them insensible to pain, has, during the past few weeks, aroused the medical profession, especially its ophthalmological branch, to a degree of interest and enthusiasm unequalled in intensity since the introduction of general anæsthesia.

A local anæsthetic has been a great desideratum from the earliest history of surgical practice to the present time, and has been sought with poor success. Various appliances, agencies and drugs have been recommended and tried, such as heat, cold and caustics, but all have been so irritating and destructive that they have, at most, received very meagre and doubtful favor. With the promise, then,—aye, with the acquisition of an agent that will answer, in a measure, at least, the great desire of ages in producing a local anæsthesia of even a part of the tissues of the body, no irritation, inflammation or pain following its use, naught could be expected from medical men but the most eager attention and interest, leading to great activity of experiment and the up-lifting of many voices in its praise.

It was on the 15th of September last, that Dr. Charles Koller, a young physician of Vienna, through Dr. Brettauer, of Trieste, presented to the Heidelberg Ophthalmological Congress a short paper on "The Local Anæsthesia of the Cornea and Globe by Means of Cocaine." Following the reading of the paper, the properties of the cocaine hydrochlorate were demonstrated upon the eyes of patients from Prof. Becker's hospital. Various reports of the congress were sent to different parts of the world, and each referred especially to Dr. Koller's observations and the cocaine experiments. Dr. H. D. Noyes, who was in attendance, was the first to herald the new discovery to the American profession through a communication to the *New York Medical Record*, which was published in its issue of October 11th. Dr. Noyes, at the same time, addressed a letter to Dr. Edward R. Squibb, of Brooklyn, N. Y., and asked him to place some cocaine hydrochlorate in the hands of Drs. C. S. Bull, of New York, and A. Mathewson, of Brooklyn. Dr. Mathewson was out of town. Dr. Bull received a sample of the preparation on October 7th, and on the following day, October 8th, used it upon the eye for the first time in America, producing anæsthesia of the cornea and removing a cinder which had become imbedded in it.

When Dr. Koller's brief, but immortal, paper was read, only two weeks had elapsed since he began his observations with cocaine hydrochlorate. He was led to make his experiments by a knowledge of its benumbing effects upon the tongue and mouth, and of its application, during the past year, by laryngologists to the vocal cords and contiguous parts, whereby these were so deprived of their sensibility that they could be handled and treated with comparative ease and comfort.

It appears that Dr. Koller began his experiments in the laboratory of Prof. Stricker. He used a solution of the same strength as that which laryngologists had used, namely, two per cent., and applied it first to the eyes of animals. He found that when dropped or brushed upon the cornea and conjunctiva it soon produced such a degree of insensibility of these structures that they could be pinched, scratched, torn or cut without the animals showing evidence of pain. He also noticed that no irritation was caused by the drug itself. He next applied it to his own eyes, and afterwards to those of other persons. The results of his brief experience, as given in his paper, may be thus summarized: 1. The strength of the solution was two per cent. 2. A few minims of this dropped into the conjunctival sac rendered the cornea and conjunctiva completely anæsthetic in from ten to fifteen minutes. 3. The anæsthesia lasted ten minutes. 4. There was slight mydriasis, which lasted a couple of hours. 5. There was some paresis of accommodation for a short time. 6. No irritation or pain followed the applica- of the solution, except a trifling smarting or burning for the first few seconds. (See *London Ophthalmic Review* for October.)

Since Dr. Koller presented to the profession the results of his experiments, Dr. Kœnigstein has come forward with claims to priority in the discovery of the properties of this drug. He stated, at a meeting of the Society of Physicians of Vienna, held October 17th, that simultaneously with, but independently of Dr. Koller, he had made experiments with cocaine applied to the eye, using a one per cent. solution of the hydrochlorate, and with much the same results as those obtained by Dr. Koller. He began his experiments towards the end of August at the suggestion of Dr. Freud, making them first upon himself and the members of his family, then upon his acquaintances, and subsequently upon a number of patients in the ward of Dr. Scholz in the Vienna General Hospital. (See *London Medical Times* for Nov. 8th.)

Upon Dr. Koller, however, must rest the honor of the discovery of the cocaine anæsthesia of the cornea and conjunctiva, and of first directing the attention of the medical profession to it.

As soon as Dr. Noyes' communication was published in the *Medical Record*, I at once procured a very small quantity of a two per cent. solution of the cocaine hydrochlorate from New York, and with it I was enabled to perform several operations upon the eye and ear without pain, a few drops having been applied to the parts two or three times at intervals of five minutes. A short time afterwards, having received a larger supply of a four per cent. solution, together with a quantity of the crystals, I had the pleasure of demonstrating, clinically, its anæsthetic properties by performing several operations painlessly upon the eye, before the medical class of Niagara University and a number of medical gentlemen whom I had invited to be present. The operations at this time included excision of a pterygium with a closure of the conjunctival wound with three sutures, tenotomy of the internal rectus muscle for convergent squint in a boy twelve years old, and two Bowman's operations (slitting up the lower lachrymal canaliculus), followed by probing of the lachrymal passages in a nervous old Irish lady. Each patient declared that there was no pain in the operation, and both spectators, patients and operator were delighted with the unmistakable and positive anæsthetic effects of the cocaine hydrochlorate there exhibited.

I have now performed a large number of operations upon the eyes of patients under its influence, using, for the most part, a four per cent. solution, instilling a drop or two of it every five minutes, till three applications have been made, when, in five minutes more, anæsthesia of the cornea and conjunctiva has generally been complete. In some patients, two applications have been sufficient, while in two or three the fourth one has seemed necessary. No unpleasant feeling attends the instillations, except occasionally a slight smarting or burning for a few seconds, and the anæsthesia lasts about fifteen to twenty minutes.

My patients have varied in age from ten to seventy-nine years, and in temperament from mild and quiet to very nervous and excitable. Many of the operations have, in themselves, been painful. But, with the aid of the local anæsthesia, I have conducted them, in every instance, without being disconcerted in any manner by the patient and without inflicting any suffering or pain. The operations have been quite varied, and include removal of foreign bodies imbedded in the cornea, cauterizing indolent corneal ulcers, tapping the anterior chamber, performing iridectomies, manipulation by Förster's method for artificially ripening cataract, extraction of senile cataracts, discission of congenital and secondary cataracts, strabismus operations, excision of pterygiums and closing the wounds with sutures, excision of a polypoid growth from the caruncle and semilunar fold, slitting up the lachrymal canaliculi, passing the lachrymal probes, and some other procedures, the anæsthetic in every case annihilating, entirely, the pain ordinarily present without its use. In performing Bowman's operation and probing the nasal duct, I have followed the slitting up of the canaliculus with an injection of a few drops of the solution into the lachrymal sac and pressing it as far as possible into the nasal duct. After a few minutes I have introduced large probes without distress. In strabismus operations I have carried two or three drops beneath the conjunctiva to the insertion of the muscle to be cut, after making the opening through the already anæsthetized conjunctiva.

I have used both two and four per cent. solutions, but principally the latter, since it has been more rapid and deeper in its effects. Besides the anæsthesia of the cornea and conjunctiva, I have noticed early, but not complete, dilatation of the pupil and partial loss of accomodation. I have also observed that the conjunctiva has appeared distinctly pale and anæmic, while the anæsthetic influence continued, and that it bled less than usual when cut. No irritation has attended or followed its application.

Each case in which I have used this substance has had a special interest to me; but there is such a sameness in the manifestations of its effects and properties in different persons that to relate several cases would be unprofitable. I will, however, detail a little personal experience which it has been my privilege to obtain.

On November 18th, I arose from bed in the morning with a sensation of a foreign body in my left eye. I was unable, however, to find anything, and yet I could not get relief from my distress. At about 3.30 P. M., I called on my friend, Dr. F. W. Abbott, of this city, who upon examination found a minute "speck" imbedded in the cornea near the centre of its anterior surface. He made several attempts to remove it without success, first with a sharpened stick and then with a cataract needle. The moment the cornea was touched an uncontrollable spasm of the ocular and orbicular muscles took place which entirely disconcerted his movements. There were also profuse lachrymation and a severe reflex spasm of the tympanic, Eustachian and palatal muscles, accompanied by a distressing tinnitus. After these fruitless efforts, it was suggested to try the cocaine hydrochlorate, when a couple of drops of a two per cent. solution were applied to the cornea. Immediately there was a slight burning or smarting which lasted but a few seconds. In two minutes the lachrymation, pain and spasm of the muscles of the eye and ear began to cease, and in three minutes the eye was entirely at ease in every respect. In four minutes two drops of a four per cent. were instilled, and in five, the doctor removed the foreign substance without any sensation whatever, except the *sound* of the "click" of the instrument, and the *sight* of the vibrations of the lamp-light concentrated upon the cornea with a lens. Neither was there any lachrymation or reflex spasm.

In about seven minutes after the instillation the pupil began to dilate, and the accommodation began at the same time to diminish. In twenty minutes the pupil was dilated to about two-thirds its full extent, remaining in this state and not contractile on exposure to light for four hours, when it commenced to diminish in size and was nearly normal in eight hours. The accommodation was affected but little; began to return in two hours and had nearly regained its full power in six hours.

In a few cases I have used the cocaine hydrochlorate, four per cent. solution, in the ear. One case was that of a polypus, which I removed painlessly. In most instances the instillations into the auditory canal have been for aural pain and tinnitus, in which, however, the results have been negative. But in removing polypi, applying caustics, incising the membrana tympani, etc., I have no doubt that it will prove equally as potent in saving pain as in operations upon the eye.

When applied to the mucous membrane of the throat and nose, I find the anæsthetic effects positive and pronounced.

With such an interesting and satisfactory experience with this substance, I can but speak of it in terms of highest praise. I find it of the utmost service in nearly all operations upon the eye and in many upon the nose, throat and ear. It is *pre-eminently useful in cataract extractions*, as the absence of pain enables the patient to submit to the operation without agitation or movement, and there is no danger of rupture of the vitreous from the vomiting which might follow the administration of chloroform or ether.

The forms in which cocaine may be used may vary. Thus far the solution of the hydrochlorate has been principally employed, but solutions of its other salts are said to be equally as good. I have not yet known of triturations being prepared, but see no reason why these may not be eligible under certain circumstances. Dr. Squibb has recently suggested that it may also be used in the form of an oleate, as the cocaine readily unites with "oleic acid and forms a true salt." The facility with which the oleates are absorbed by the skin and the depth to which they may possibly penetrate, give, theoretically, great promise for the use of such a preparation in the relief of local pain. (*Ephemeris* for November.)

The strength of the preparations may range from two to twenty per cent. and may need to be varied according to certain peculiarities of the parts, or conditions or idiosyncrasies of patients. I have found, as already stated, a four per cent. solution suitable for anæsthesia of the cornea and conjunctiva.

The solutions should also be fresh, as the anæsthetic properties seem to be destroyed by very slight changes. A four per cent. solution in my possession became so changed at the end of three weeks that I was unable to produce anæsthesia of the cornea and conjunctiva with it, although it dilated the pupil early and rapidly as before. This failure occurred in several cases ; but in the first one it was particularly annoying. It was the case of a boy thirteen years of age on whom I was about to do a second operation for discission of congenital cataract. I visited the patient at his home and carried with me only the old solution which I had previously used upon him and upon others with success. On instilling it into his eye, he complained of considerable smarting, but I paid little attention to it and continued the instillations every five minutes as usual. At the end of fifteen minutes I tested the sensibility of the conjunctiva and found it was not diminished. The solution was continued freely every five minutes for half an hour, but without any anæsthesia whatever. I then postponed the operation for twenty-four hours, when I applied to the eye a fresh solution of the same strength, twice, at an interval of five minutes. At the end of five minutes more the cornea and conjunctiva were examined and found insensible. The third instillation was then added to make "surety doubly sure," and in another five minutes I proceeded to incise the anterior surface of the lens, the child declaring that he "did not feel anything."

On examining the old solution, it was "musty" to the smell, turbid in looks, and, under the microscope, showed fungous growths and other evidence of decomposition.

The failures of an old solution to produce anæsthesia, as with a fresh preparation, indicate the instability of the anæsthetic properties of cocaine, and show how easily they are lost, although its mydriatic action remains. Is it not possible that the reported failures to obtain the anæsthesia may be attributed to decomposition or other changes of the cocaine solution?

Cocaine preparations may, possibly, be protected from decomposition and change by incorporating some antiseptic. Dr. Squibb suggests preparing solutions of any desired strength with equal parts of water and a cold saturated solution of salicylic acid, which will, he thinks, remain clear and free from microscopic organisms for an indefinite period of time. The solutions so protected he believes may be used without causing perceptible irritation.

Solutions may be applied in different ways, using a brush, a pipette, a syringe, paper or other material saturated with it, or, what seems to me most suitable for the eye, ear, nose and throat, a small and delicate spray, and especially since its high price makes economy in its use a matter of consideration.

My own interesting and most satisfactory experience with cocaine hydrochlorate, both upon myself and my patients, permits me to speak of it only in terms of confidence and commendation. I would not, however, lead my readers to believe that I can see only a bright side to its use, for I do recognize a dark side. In the first place, it may affect different persons in a different manner, possibly failing entirely in some. Secondly, when taken into the system in too large quantities it may produce unpleasant and toxic effects.

Just how much cocaine is necessary to produce toxic symptoms has not yet been fully determined. According to some experiments by Dr. R. J. Hall, of New York, upon himself (*New York Medical Journal* for December 6th), twenty minims of a four per cent. solution of the hydrochlorate (about four-fifths of a grain), could be taken hypodermically without constitutional effects; but a hypodermic injection of thirty-two minims of the same solution (about one and one-fourth grains), induced giddiness to the extent of his being unable to walk without staggering, quite severe nausea, cold perspiration and dilatation of the pupils. The nausea passed off in about twenty minutes, and the dizziness in about an hour and a half. The possible unpleasant effects of doses of more than one grain must, therefore, be borne in mind.

The experience of myself and that recorded by others has not only most fully corroborated the results of Dr. Koller's original observations, but it has also extended the range of application of cocaine anæsthesia far beyond the cornea and conjunctiva, till it is now used upon denuded surfaces and mucous membranes of all accessible parts of the body, and is also carried through wounds and by the hypodermic syringe deeply into tissues beneath the surface. When thus introduced beneath the skin, it destroys the sensibility of all the parts with which it comes in contact, and when it reaches the trunk of a sensitive nerve the branches of it given off beyond the point of contact are rendered more or less completely anæsthetic.

Thus cocaine may be made to reach and affect many parts of the body, and may be put to an infinite variety of uses. It may be used to allay the pain of many diseases and operations, and to assuage the throes of certain stages of child-birth by applying it to the perineum and labiæ. By using it hypodermically, even quite formidable operations can be executed with little suffering, the cocaine being thus applied directly to the parts to be cut. In this way, the eye-ball can be enucleated, tumors removed, and a variety of operations performed, it being only necessary to remember to restrict the amount introduced within the limits of safe dosage.

Since the time of the first discovery of an alkaloid of the coca-leaf (erythroxylon coca), by Gædeke, in 1855, and independently by Dr. Samuel R. Percy, of New York, in 1857, which both named "erythroxyline," and again extracted and studied by Albert Nieman, of Goslar, Germany, in 1860, who gave it the name "cocaine," this substance and all its salts have been regarded principally as chemical and pharmaceutical curiosities and treated with great indifference by the profession. But suddenly, through the wit of a Vienna medical gentleman, it rapidly attains a most distinguished position, and the whole medical world arises in profound astonishment and wonder at its mysterious power. Its properties are so unique, its effects so simple and yet so positive, so free from annoyances and extraneous symptoms, and its action so generally uniform in character that it at once commands the admiration and confidence of all.

All honor to Charles Koller! But why should this discovery have awaited him? The anæsthetic effects of this alkaloid were hinted at by Percy and Nieman, and by subsequent writers. Moréno y Mäiz, in 1868, and Von Anrep, in 1880, stated that cocaine injected under the skin abolished the sensibility of the part. Rossbach and Nothnagel in their *Materia Medica*, 1880. referred to its "local anæsthetic effect on mucous membranes." Von Anrep applied it to the conjunctiva and observed that it dilated the pupil, but did not notice that it diminished the sensibility of the parts. Freud had also referred to the local anæsthesia by cocaine. Certain laryngologists of the continent, acting upon the published statements of those who had studied the subject, had, during the past year, produced anæthesia of the throat. But it still remained for Koller to clearly determine and demonstrate to the medical profession its distinctive properties of anæsthesia, and for him to become the distinguished discoverer of one of the most potent agencies for the local relief of suffering and the removal of the distressing apprehensions of pain in operations that has ever been added to our materia medica. Cocaine may have other excellent virtues, but it seems to us that its anæsthetic properties alone are sufficient to place it among the first in the list of drugs powerful to bless mankind.

